# Establishing language and ethnic equivalence for health-related quality of life item banks and testing their efficiency via computerised adaptive testing simulations

**DOI:** 10.1371/journal.pone.0298141

**Published:** 2024-02-23

**Authors:** Yu Heng Kwan, Eva Fenwick, Ying Ying Leung, Warren Fong, Ting Hui Woon, Ling Xiang, Ecosse Lamoureux, Julian Thumboo

**Affiliations:** 1 Department of Rheumatology and Immunology, Singapore General Hospital, Singapore, Singapore; 2 Program in Health Services and Systems Research, Duke-NUS Medical School, Singapore, Singapore; 3 Centre of Population Health and Implementation Research, SingHealth Regional Health System, Singapore, Singapore; 4 Singapore Eye Research Institute and Singapore National Eye Centre, Singapore, Singapore; 5 The University of Melbourne, Melbourne, Australia; 6 Duke-NUS Medical School, Singapore, Singapore; Universidad Santiago de Cali, COLOMBIA

## Abstract

**Purpose:**

We aimed to (1) establish linguistic and ethnic equivalence (i.e. lack of bias) for the items in the English and Chinese versions of the Singapore Health and Well Being (SHAWS) Physical Functioning (PF), Positive Mindset (PM) and Social Relationship (SR) item banks (IBs); and (2) evaluate the preliminary efficiency of these IBs using Computer Adaptive Testing (CAT) simulations.

**Methods:**

In this cross-sectional study, 671, 670, and 672 subjects answered 55, 48 and 30 items of the PF, PM, and SR IBs, respectively. Rasch analysis was conducted to assess each IB’s psychometric properties, particularly the presence of differential item functioning (DIF) for language and ethnicity. A set of performance criteria related to removing items that displayed notable DIF were employed. CAT simulations determined the mean number of items for high, moderate, and moderate-low measurement precisions (stopping rule: SEM 0.300, 0.387. 0.521, respectively).

**Results:**

Half of subjects were >50 years old (40.9% PF, 42.1% PM, 41.4% SR), Chinese (50.7% PF, 51.0% PM, 50.6% SR) and female (50.0% PF. 49.4% PM, 52.8% SR) respectively. Rasch analysis revealed 4 items with DIF for the PF IB, 9 items with DIF for the PM IB and 2 items with DIF for the SR IB. In CAT simulations, the mean number of items administered was 8.5, 21.6 and 14.5 for the PF, PM and SR IBs, respectively (SEM 0.300), 5.1, 13.0, 8.0 for PF, PM and SR IBs, respectively (SEM 0.387) and 3.1, 5.3 and 4.1 for PF, PM and SR IBs, respectively (SEM 0.521).

**Conclusion:**

The PF, PM and SR IBs to measure health-related quality of life revealed minimal DIF for language and ethnicity after remedial efforts. CAT simulations demonstrated that these IBs were efficient, especially when the stopping rule was set at moderate precision, and support the implementation of the SHAWS IBs into routine clinical care.

## Introduction

As emphasised by the World Health Organisation (WHO), health is a state of complete physical, mental and social well-being and not merely the absence of disease or infirmity [1]. In line with this approach, we have previously shown that physical functioning (PF), positive mindset (PM) and social relationship (SR) are the domains most relevant to health-related quality of life (HRQoL) in Singapore [2]. Being able to measure these domains will allow appropriate interventions designed to improve HRQoL [3]. Although there are numerous fixed-length patient-reported outcome measures (PROMs) that measure generic HRQoL, their uptake in clinical practice has been limited, in part because they are lengthy and burdensome to complete as every item must be administered regardless of whether the difficulty level of an item is relevant for a given patient [4].

Computerised adaptive testing (CAT) presents a novel solution to improve the uptake of PROM administration in clinical practice by reducing the length and respondent burden of these measures [4]. Item banks (IBs) are repositories of items that have been calibrated using item response theory (IRT) or Rasch analysis, while CATs use the IB items to tailor each test to the participants [5]. CATs allow reductions in questionnaire burden as they adapt the questions based on what the respondent has answered previously, thus allowing a more precise measurement of the latent construct with fewer questions than a traditional fixed-length questionnaire [6]. CAT administration uses an algorithm to match participants to the most informative items within a PROM and, once an acceptable level of precision is reached, no further items are required. As part of their development, CAT simulations are run to test the efficiency of the IBs, i.e. how many items needed to provide estimates of the latent trait at predetermined levels of precision.

When using CATs in multi-ethnic contexts, the impact of language of administration and ethnicity needs to be assessed and addressed. It is important that there is minimal or acceptable differential item functioning (DIF). DIF indicates if item bias is present for certain participant characteristics, such as ethnicity and language of administration [7]. This is important as evidence suggests that different lifestyle behaviours across ethnicities may result in DIF when administering multi-item construct questionnaires [8, 9]. Having an IB free of DIF allows for comparison of scores between different ethnicities and languages [10] and is therefore important to bring the IBs to routine clinical implementation [11]. Recognizing the potential impact of language of administration and ethnicity, we have concurrently developed English and Chinese language versions of the SHAWS PF, PM and SR IBs in Singaporean community-dwelling adults [2, 12–15]. However, an assessment of DIF for language or ethnicity of the English and Chinese versions of these IBs has not been conducted, nor has the efficiency of our IBs been evaluated. Ensuring that items in an IBs are free from ethnic and/or linguistic bias is an important but often overlooked aspect of PROM development and can hinder implementation of PROMs in clinical care, especially in societies that are multilingual such as Singapore [16].

Therefore, we aimed to (1) investigate the presence of DIF for language and ethnicity in English and Chinese versions of the SHAWS PF, PM and SR IBs and to address the presence of DIF by removing or retaining items; and (2) evaluate the efficiency of the final calibrated IBs using a CAT simulation application.

## Methods

### Study design

This study extends our previous work reporting the development of IBs measuring PF, PM and SR [12]. In the current study, we focused specifically on the methodology and results for testing linguistic and ethnic DIF for each item bank, and the evaluation of IBs’ efficiency using CAT simulations. This study was approved by the SingHealth Centralized Institutional Review Board (Ref 2016/2337) and all participants signed written informed consent. The study was conducted according to the guidelines of the Declaration of Helsinki.

### Subject recruitment and data collection

We recruited Singapore citizens or permanent residents from the community and the Singapore General Hospital from 13 September 2016 to 27 December 2016. We sampled 75% English and 25% Mandarin speaking subjects separately. Within each language sampling frame, we used purposive sampling to select subjects based on age, gender, ethnicity and presence of chronic illnesses. **S1 Table** provides the list of chronic illnesses based on the Singapore Burden of Disease study [17]. Subjects were classified as well, mildly unwell and unwell based on the number and severity of chronic illnesses. We excluded subjects who were unable to have a meaningful discussion with our interviewers, due to mental illnesses or cognitive impairment. In order to include subjects from a wide spectrum of health, we predefined the proportion of subjects to be recruited as 35% well, 15% mildly unwell and 50% unwell.

Subjects from the community were recruited by a survey company from a residential household sampling frame of public housing, in which more than 80% of Singaporeans reside. The primary sampling unit were plots of land with approximately equal numbers of households, stratified according to geographic location and dwelling type. Households in each primary sampling unit were selected based on fixed route rules and skip patterns based on pre-specified ethnic and age quotas. Only one respondent per household was selected to participate in the study. Three call attempts were made at different times of the day with at least 1 call on a non-work day (Saturday or Sunday) to improve the response rate. This sampling method has been used in the Singapore Health Survey [18]. The response rate of the survey was computed using the standard set by the Council of American Survey Research Organization [19], which is defined as the number of completed interviews divided by the number of eligible reporting units in sample. Assessments were completed on-site at subjects’ home. The subject was reimbursed for their time on completion of the interview.

Interviewers administered the SHAWS items, with each participant completing the questions from the either the PF, PM or SR IBs [2]. Interviewers administered the survey so that illiterate subjects could be included. The PF IB measures physical functioning and comprised 55 items, each with a 5-level response option adapted from Patient-Reported Outcomes Measurement Information System (PROMIS). The PM IB measures the positive mindset of a subject and comprised of 48 items while the SR IB measures the depth and meaningfulness of the human connections the subject has and comprised of 30 items [12, 14]. The response options were “Never”, “Seldom”, “Sometimes”, “Usually” and “Always” for items on frequency and “Not at all”, “Mildly”, “Moderately”, “Quite a lot” and “Extremely” for items on intensity [15].

Demographics including age, gender, ethnicity, education and current medical status and the participant-reported global assessment of health were also collected.

### Assessment of DIF for language and ethnicity of the PF, PM and SR IBs

Each IB was analyzed using Rasch analysis with Winsteps 4.50 software (Winsteps, Beaverton, OR) using the Andrich single-rating scale model [20]. Rasch analysis estimates the relative difficulty of items (item measures) and relative abilities of respondents (person measures) and aligns them on an interval level scale, and these item calibrations are used for the CAT algorithm. Rasch analysis also provides substantial information about a scale’s psychometric properties, including assessment of DIF. We took a DIF contrast of ≥0.64 logits with a corresponding significant Rasch-Welch probability [21]. Bonferroni correction was applied for multiple DIF tests (p-value / no. items) [22].

Through DIF assessment, we identified items with DIF for ethnicity (Chinese, Malay, Indian) and/or language (English, Chinese) and considered them for removal. Item removal was indicated if 1) the DIF was particularly problematic (i.e., DIF contrast [DC] substantially greater than ±0.64 logits after Bonferroni correction), 2) item content was deemed non-essential or covered by other similar items, and/or 3) there were obvious reasons for DIF to occur, i.e., culturally specific activity). All decisions involving item deletion were reviewed and approved by the research team, comprised of members with content development and psychometric expertise (EF, EL, JT) and/or clinical expertise (JT).

### Assessment of the efficiency of the item banks using CAT simulations

CATs for the PF, PM and SR IBs were developed and hosted on an online CAT testing platform (PROMinsight) using Concerto open-source software [23]. We then conducted simulations to assess the efficiency of our Winsteps threshold calibrations (JMLE; Joint Maximum Likelihood Estimation method) and associated CAT algorithm [24] in 1000 simulated respondents using R Statistical Computing Environment [25]. Individual packages were loaded in R to conduct IRT including CAT simulations (“catR”) [26]. Simulations were based on a standard normal distribution (M = 0, SD = 1) and used the Rating-Scale Model (RSM), the ML (maximum likelihood) estimator and the Maximum Fisher Information (MFI) item selection criteria. We determined the average number of items required based on three different stopping rules: SEM of 0.30 representing “high precision”, 0.387 representing “moderate precision”, and 0.521 representing “moderate-low precision”. High precision (reliability ~0.91) may be appropriate for high-stakes testing (e.g. clinical trials); moderate precision (reliability ~0.85) and moderate-low precision (reliability ~0.73) may be appropriate for lower-stakes testing such as routine clinical monitoring of patients and where testing brevity is important [27]. Model fit was assessed using the root mean square error (RMSE) and level of bias between true and estimated ability levels (low values are desirable). We also calculated the Pearson correlation coefficient between the IBs and CAT simulated person measure estimates. We hypothesized high (r ≥ 0.85) and moderate-high (0.75 ≥ r < 0.85) correlations for simulations with the high and moderate precision stopping rules, respectively. Results across the spectrum of the latent trait are summarized in **Table 3**. Results for specific ‘ability’ levels are provided in deciles (D1-D10 n = 100 each, where D1 and D10 includes simulees at the lowest and highest ‘ability’ levels, respectively; **S2–S4 Tables**).

## Results

### Demographics

**Table 1** summarizes the participants’ demographics of the SHAWS study. Out of 2013 subjects, 1492 (74.1%) and 521 (25.9%) completed the English and Chinese versions, respectively. The majority of recruited subjects were Chinese (50.8%), females (51.0%) and had completed at least 10 years of education (i.e. secondary school) (76.5%). The number of subjects who completed the PF, PM and SR IBs were largely equal [PF:PM:SR (n = 671, 670, 672 respectively)].

**Table 1:** Sociodemographic characteristics of the study subjects.

	Frequency (%)			
	Total	PF	PM	SR
	N = 2013	N = 671	N = 670	N = 672
Age				
21–49 years old	965 (47.9)	326 (48.6)	317 (47.3)	322 (47.9)
50 and above	1048 (52.1)	345 (51.4)	353 (52.7)	350 (52.1)
Gender				
Male	986 (49.0)	334 (49.8)	330 (49.3)	322 (47.9)
Female	1027 (51.0)	337 (50.2)	340 (50.7)	350 (52.1)
Language interviewed				
English	1492 (74.1)	496 (73.9)	493 (73.6)	503 (74.9)
Chinese	521 (25.9)	175 (26.1)	177 (26.4)	169 (25.1)
Ethnic group				
Chinese	1022 (50.8)	340 (50.7)	342 (51.0)	340 (50.6)
Malay	492 (24.4)	162 (24.1)	161 (24.0)	169 (25.2)
Indian	499 (24.8)	169 (25.2)	167 (25.0)	163 (24.2)
Employment status				
Currently working	1211 (60.2)	429 (63.9)	382 (57.0)	400 (59.5)
Currently not working	802 (39.8)	242 (36.1)	288 (43.0)	272 (40.5)
Completion of secondary education (10 years of education)				
Yes	1539 (76.5)	511 (76.1)	503 (75.1)	525 (78.1)
No	474 (23.5)	160 (23.9)	167 (24.9)	147 (21.9)

Abbreviations: Physical Functioning (PF), Positive Mindset (PM), Social Relationship (SR)

### DIF assessment for the three IBs

Initial Rasch analysis of the PF IB revealed two items with DIF for ethnicity and language (**Table 2**). The first was item 15 “I am able to use a pair of chopsticks” (DC -2.23 Chinese-Malay and -1.94 Chinese Indian) indicating that Chinese respondents found this item easier to do than their Malay and Indian counterparts, regardless of their underlying level of physical functioning. The second was item 25 “I am able to roll onto my stomach, while lying in bed, without assistance from other people” which was significantly easier for Chinese participants compared to Malays (DC -0.97) and Indians (DC -0.99). We found that two items were harder to endorse for the English version compared to the Chinese version of the IB (DC 2.07 and 1.30, respectively), with this difference unrelated to their underlying level of physical function. In addition, item 42 “I am able to run a full marathon (42 kilometers)” displayed DIF for ethnicity, while item 61 “I am bedridden” displayed DIF for language (**Table 2**). Upon deletion of items 15 and 25, DIF for item 61 was no longer apparent, DIF for ethnicity for item 42 remained, and DIF for ethnicity for item 45 “I am able to carry 2 bags filled with groceries” had emerged, with Chinese people finding this item harder to do than Malays (DC 0.71). Because the DCs marginally exceeded the cut-off value of 0.64 logits for items 42 and 45, and the content was deemed to be important and not covered by other items, these two items were retained.

**Table 2:** Differential item functioning (DIF) for ethnicity and language for the physical functioning, positive mindset, and social relationship item banks.

Item no., content	DIF type	DIF contrast^a^^,^^b^	Groups affected	Interpretation	Removed
*Physical functioning*					
15, I am able to use a pair of chopsticks	Ethnicity	-2.23	Chinese-Malay Chinese-Indian	Chinese respondents found this item easier to perform than their Malay and Indian counterparts regardless of their underlying level of physical functioning	Yes
		-1.94			
15, I am able to use a pair of chopsticks	Language	2.07	English-Chinese versions	Respondents of English version found this item harder to endorse than respondents of Chinese version regardless of their underlying level of physical functioning	Yes
25, I am able to roll onto my stomach, while lying in bed, without assistance from other people	Ethnicity	-0.97	Chinese-Malay Chinese-Indian	Chinese respondents found this item easier to perform than their Malay and Indian counterparts regardless of their underlying level of physical functioning	Yes
		-0.99			
25, I am able to roll onto my stomach, while lying in bed, without assistance from other people	Language	1.30	English-Chinese versions	Respondents of English version found this item harder to endorse than respondents of Chinese version regardless of their underlying level of physical functioning	Yes
42, I am able to run a full marathon (42 kilometers)	Ethnicity	-0.85	Chinese-Malay	Chinese respondents found this item easier to perform than their Malay counterparts regardless of their underlying level of physical functioning	No
45, I am able to carry 2 bags filled with groceries	Ethnicity	0.71	Chinese-Malay	Chinese respondents found this item harder to perform than their Malay counterparts regardless of their underlying level of physical functioning	No
61, I am bedridden	Language	-1.01	English-Chinese versions	Respondents of English version found this item easier to endorse than respondents of Chinese version regardless of their underlying level of physical functioning	No
*Positive mindset*					
1, I am able to accept people as they are	Language	-0.73	English-Chinese versions	Respondents of English version found this item easier to endorse than respondents of Chinese version regardless of their underlying level of positive mindset	Yes
5, I am able to appreciate the people in my life	Language	0.79	English-Chinese versions	Respondents of English version found this item harder to endorse than respondents of Chinese version regardless of their underlying level of positive mindset	No
24, I enjoy life	Ethnicity	0.80	Chinese-Malay	Chinese respondents found this item harder to endorse than their Malay counterparts regardless of their underlying level of positive mindset	No
25, I find comfort in my religion or spiritual beliefs	Ethnicity	1.75	Chinese-Malay Chinese-Indian	Chinese respondents found this item harder to endorse than their Malay and Indian counterparts regardless of their underlying level of positive mindset	Yes
		1.35			
25, I find comfort in my religion or spiritual beliefs	Language	-1.13	English-Chinese versions	Respondents of English version found this item easier to endorse than respondents of Chinese version regardless of their underlying level of positive mindset	Yes
26, I find comfort in my religious beliefs	Ethnicity	1.86	Chinese-Malay Chinese-Indian	Chinese respondents found this item harder to endorse than their Malay and Indian counterparts regardless of their underlying level of positive mindset	Yes
		1.36			
26, I find comfort in my religious beliefs	Language	-1.17	English-Chinese versions	Respondents of English version found this item easier to endorse than respondents of Chinese version regardless of their underlying level of positive mindset	Yes
35, I worry about not being accepted by others	Ethnicity	-1.18	Chinese-Malay Chinese-Indian	Chinese respondents found this item easier to endorse than their Malay and Indian counterparts regardless of their underlying level of positive mindset	Yes
		-0.86			
35, I worry about not being accepted by others	Language	0.85	English-Chinese versions	Respondents of English version found this item harder to endorse than respondents of Chinese version regardless of their underlying level of positive mindset	Yes
36, I am easily discouraged	Ethnicity	-0.91	Chinese-Malay	Chinese respondents found this item easier to endorse than their Malay counterparts regardless of their underlying level of positive mindset	Yes
36, I am easily discouraged	Language	0.68	English-Chinese versions	Respondents of English version found this item harder to endorse than respondents of Chinese version regardless of their underlying level of positive mindset	Yes
37, I am bothered by small matters	Ethnicity	-0.86	Chinese-Malay	Chinese respondents found this item easier to endorse than their Malay counterparts regardless of their underlying level of positive mindset	Yes
37, I am bothered by small matters	Language	0.65	English-Chinese versions	Respondents of English version found this item harder to endorse than respondents of Chinese version regardless of their underlying level of positive mindset	Yes
38, I tend to overthink things	Ethnicity	-0.72	Chinese-Malay	Chinese respondents found this item easier to endorse than their Malay counterparts regardless of their underlying level of positive mindset	Yes
38, I tend to overthink things	Language	0.71	English-Chinese versions	Respondents of English version found this item harder to endorse than respondents of Chinese version regardless of their underlying level of positive mindset	Yes
39, I give up easily	Ethnicity	-0.97	Chinese-Malay	Chinese respondents found this item easier to endorse than their Malay counterparts regardless of their underlying level of positive mindset	Yes
40, I lose my temper easily	Ethnicity	-0.66	Chinese-Malay	Chinese respondents found this item easier to endorse than their Malay counterparts regardless of their underlying level of positive mindset	Yes
46, I take pride in my achievements	Language	-0.67	English-Chinese versions	Respondents of English version found this item easier to endorse than respondents of Chinese version regardless of their underlying level of positive mindset	No
48, I am not a bad person	Language	-0.67	English-Chinese versions	Respondents of English version found this item easier to endorse than respondents of Chinese version regardless of their underlying level of positive mindset	Yes
*Social Relationship*					
48, Overall, I am satisfied with the support I get from my friends	Ethnicity	-0.76	Chinese-Indian	Chinese respondents found this item easier to endorse than their Indian counterparts regardless of their underlying level of social relationship	No
50, Overall, I am satisfied with the support I give to others	Language	0.75	English-Chinese versions	Respondents of English version found this item harder to endorse than respondents of Chinese version regardless of their underlying level of social relationship	No

^a^A *positive* DIF contrast means the item is *more* difficult for the reference group (Chinese); while a *negative* DIF contrast means the item is *less* difficult for reference group (English-speakers).

^b^DIF contrast of ≥0.64 was considered as moderate-large [1]. Bonferroni correction was applied for multiple DIF tests (p-value / no. items) [2] = e.g. 0.05/54 = 0.0009.

Initial Rasch analysis of the PM IB revealed six items with both DIF for ethnicity and language (items 25, 26, 35–38), two items with DIF for ethnicity (items 39, 40), and two items with DIF for language (item 1 and 48; **Table 2**). These items also displayed substantial misfit (high noise-to-signal ratio) and were iteratively deleted to resolve the misfit and DIF. Following this, DIF emerged for language for items 5 and 46 and for ethnicity for item 24; however, these items were retained as their DC marginally exceeded the target cut-off and content was considered important by the study team.

For the SR IB, Rasch analysis revealed two items with DIF (**Table 2**), one each for language (item 50) and ethnicity (item 48). However, as their DC values marginally exceeded the target cut-off and item content was felt to be important, they were retained.

### CAT Simulations

Overall, a good model fit (low RMSE and bias values) was achieved for all simulations (**Table 3**). With an 0.3 SEM stopping rule, the mean number of items administered across the spectrum of the latent trait was 8.5, 21.6 and 14.5 for the PF, PM, and SR IBs, respectively (**Table 3**), with the proportion of participants satisfying the stopping rule ranging between 89% (PM) to 99.6% (PF). For ‘moderate’ precision (stopping rule SEM 0.387), the mean number of items administered across the spectrum of the latent trait was 5.1 for PF, 13.0 for PM and 8.0 for SR, with 100% of participants meeting the stopping rule for all three IBs (**Table 3**). Finally, when the stopping rule was SEM 0.521 (‘moderate-low precision’), the mean number of items administered was very low (3.1 for PF, 5.3 for PM and 4.1 for SR) and 100% of participants met the stopping rule for all three IBs (**Table 3**). When looking at specific participant ‘ability’ levels, the mean number of items required was generally lowest for participants in the middle deciles (i.e., D3-D8) and highest for those at the very ‘unable’ (D1-D2) and very ‘able’ (D9-D10) ends of the ability spectrum (**S2–S4 Tables**).

**Table 3:** CAT simulation results for the physical function, positive mindset, and social relationship item banks.

*SEM 0*.*3 (high precision)*					
**Domain, no. items**	**Mean no. items**	**Correlation with full IB person measure**	**RMSE**	**Bias**	**% satisfied stopping rule**
PF, 54	8.5	0.85	0.58	-0.07	99.6%
PM, 37	21.6	0.73	0.88	-0.13	89.0%
SR, 44	14.5	0.74	0.90	-0.13	99.6%
*SEM 0*.*387 (moderate precision)*					
PF, 54	5.1	0.84	0.57	-0.09	100%
PM, 37	13.0	0.80	0.82	-0.16	100%
SR, 44	8.0	0.75	0.85	-0.15	100%
*SEM 0*.*521 (moderate-low precision)*					
PF, 54	3.1	0.83	0.58	-0.06	100%
PM, 37	5.3	0.73	0.77	-0.14	100%
SR, 44	4.1	0.75	0.74	-0.13	100%

CAT = Computerized adaptive testing; IB = Item bank PF = Physical function; PM = Positive mindset; SR = Social relationships; RMSE = Root mean square error; SEM = Standard error of measurement

Correlations between person measures generated by the CATs compared to the full IBs were moderate to high, ranging between 0.73–0.85 for SEM 0.3, 0.75–0.84 for SEM 0.387 and 0.73–0.83 for SEM 0.521.

## Discussions

In this large community-based study of participants answering the 3 SHAWS IBs, we found some initial evidence of DIF (item bias) for language and ethnicity, with certain subgroups finding items harder or easier to endorse than their counterparts despite having similar levels of HRQoL. Using a systematic set of criteria, remedial action was taken to resolve the DIF by deleting particularly problematic items while retaining others that were of borderline concern but important for content validity. Following this process, language (English/Chinese) and ethnic equivalence was demonstrated for the SHAWS PF, PM and SR IBs, supporting their use to allow uniform measurement of HRQoL in the clinical setting. Our CAT simulation results were promising, with few items needed to provide robust measurement of PF, PM and SR, further supporting their implementation in routine clinical practice and in clinical trials.

After a thorough investigation using Rasch analysis, multiple items were identified as having notable DIF (item bias) for language or ethnicity. Most were removed to ensure that item calibrations were free from bias; however, 7 items were retained (2 for PF, 3 for PM and 2 for SR) because the DC values only just surpassed the target cut-off and, after consideration from the study team, item content was deemed to be important. Based on the guidelines provided by Teresi et al, small level of DIF is tolerable in an item bank if the items with DIF are felt to be clinically important [28]. Our study is similar to that of Kong et al whereby they showed that DIF for the English and Chinese version of the Systemic Lupus Erythematosus Quality of Life was largely not significant [29]. Another study by Lau et al has also demonstrated limited influence of DIF on the Taiwanese Chinese and Canadian English versions of the PhoPhiKat-45 scale despite differences in ethnicity and languages of the respondents [30]. Our results and these studies provide a firm basis for the implementation of the SHAWS IBs into routine clinical practice.

Overall, our simulation results were promising, particularly for our moderate precision stopping rule target where CAT administration could reduce the number of items by our 3 IBs needed by 65–90% (depending on the domain) compared to the full IB. CAT efficiency was reduced with the high precision stopping rule for PM and SR, where the number of items needing to be administered was 21.6 and 14.5 respectively. Generally, measurement was most efficient and precise for patients in the middle of ‘ability’ level for each construct at each stopping rule, and least efficient (required more items) and precise (had a larger SEM) at the lowest (i.e. D1-2) and highest (D9-10) end of the ability spectrum. This is likely because less items were available to target those with the highest and lowest ability levels and, consequently, the CAT algorithm was limited in the items which it could select. Future work aims to replenish the IBs with items targeting the more extreme ends of the spectrum. Addition of novel items to IBs is possible using Rasch analysis by estimating the calibration of new items relative to existing ones [31]. While users should be aware that the 3 HRQoL IBs were more limited in providing stable ability estimates in very ‘able’ patients, poor targeting in the upper score range is not necessarily problematic in healthcare, as clinicians may not focus on disease and treatment monitoring in patients with few HRQOL issues. However, clinicians should be aware that score estimates may not be as precise for those at the very ‘unable’ end of the spectrum and that more items may be required to properly assess this subset of the population. Overall, our results support the application of CAT over fixed-length questionnaires in assessing HRQoL in routine clinical care due to reductions in time and administrative burden, similar to other studies [32].

Correlations between the CAT and full IB person measures were somewhat lower than expected (~ 75–80% compared to the expected ≥ 0.85) and also lower than that obtained by PROMIS CATs in other health conditions (> 0.90) [33, 34], suggesting that potential loss of accuracy in scores may accompany the reduction in response burden offered by the 3 HRQOL IBs. This is particularly relevant for individual level comparisons, where reliabilities ≥ 0.90 are usually required. Future work will focus on conducting a formal comparison between CAT and full IB person measures using real data, which will provide a better understanding of the effectiveness of the PF, SR, and PM IBs in reducing response burden while replicating full IB scores.

### Strengths and limitations

Our study has several strengths. First, we had a large sample size and this is important to obtain accurate item-parameter estimates [35]. Second, we used purposive sampling to ensure applicability to age groups, gender and ethnicities, allowing a thorough investigation of ethnic and linguistic DIF. Our studies also had the following limitations. First, the IBs were not administered in Malay or Tamil which means that there may be undetected item bias for these languages. Second, we adopted an item removal approach to resolve DIF, whereas other studies have suggested managing cultural DIF using item anchoring, where parameters of items with DIF are allowed to vary, while item calibrations for the other items which displayed DIF remain constant across each country [36]. However, many of the items with DIF in our study also displayed substantial item misfit suggesting that they were not contributing to measurement and, as such, required deletion in any case. Third, the efficiency of the IBs is limited at the extremes of ability levels. We will work towards replenishing the IBs as part of our future work to better measure subjects at the very high or very low ends of the intended latent traits. Next, our data were collected pre COVID and it is possible that COVID-19 may have changed participants’ perceptions of quality of life. However, given that our IBs assess general health-related QoL and do not specifically tap into COVID-related issues, we expect the impact of COVID-19 on our IBs to be minimal. Finally, we used the standard normal distribution (M = 0, SD = 1) to run our simulations. Given that our IB calibrations were based on a relatively able participant sample with high mean person measures, simulating latent traits from a normal distribution with these parameters may have affected the accuracy of our results.

## Conclusions

After a thorough exploration of item bias and systematic remedial action to address it, we demonstrated the language and ethnicity equivalence of English and Chinese SHAWS PF, PM and SR IBs. We also demonstrated that the 3 IBs were efficient via CAT simulations, with very few items required to gain measurement of HRQoL especially at moderate precision levels. These results provide further evidence that support implementation of the PF, PM and SR IBs in routine clinical care.

## Supporting information

S1 Table Qualifying conditions for patient recruitment. (DOCX)

S2 Table Simulation results *physical functioning* at three different precision stopping rule estimates across deciles of participant ability level. (DOCX)

S3 Table Simulation results for *social relationship* at three different precision stopping rule estimates across deciles of participant ability level. (DOCX)

S4 Table Simulation results for *positive mindset* at three different precision stopping rule estimates across deciles of participant ability level. (DOCX)

S1 Data (XLS)
