# Supplementary material for: Establishing language and ethnic equivalence for health-related quality of life item banks and testing their efficiency via computerised adaptive testing simulations
Source: PLoS One. 2024 Feb 23;19(2):e0298141. doi: 10.1371/journal.pone.0298141 (PMC10890744; doi:10.1371/journal.pone.0298141)
Supplement: S1 Table — (DOCX) [file pone.0298141.s001.docx]

**S1 Table.** Qualifying conditions for patient recruitment

| **List A** | **List B** |
| --- | --- |
| - Age-related macular degeneration - Anxiety disorder - Asthma - Breast cancer - Chronic obstructive pulmonary disease (COPD) - Colon cancer - Depression - Diabetes - Diabetic retinopathy - Glaucoma - Hearing loss, adult-onset - Heart failure - Ischemic heart disease - Lung cancer - Migraine - Osteoarthritis - Rheumatoid arthritis - Stroke | - Cancer except breast or color cancer - Epilepsy - Heart arrhythmia - Heart disease except heart failure, ischemic heart disease or heart arrhythmia - High cholesterol - Hypertension - Psoriasis - Joint disease except rheumatoid arthritis or osteoarthritis - Kidney disease not requiring dialysis or transplantation - Benign prostate hyperplasia - Osteoporosis - Blindness from any cause - Cataract - Any other diseases |
