# Supplementary material for: Establishing language and ethnic equivalence for health-related quality of life item banks and testing their efficiency via computerised adaptive testing simulations
Source: PLoS One. 2024 Feb 23;19(2):e0298141. doi: 10.1371/journal.pone.0298141 (PMC10890744; doi:10.1371/journal.pone.0298141)
Supplement: S2 Table — (DOCX) [file pone.0298141.s002.docx]

| **S2 Table.** Simulation results ***Physical Functioning*** at three different precision stopping rule estimates across deciles of participant ability level | | | | | | | | | | |
| --- | --- | --- | --- | --- | --- | --- | --- | --- | --- | --- |
| **Results at SEM 0.30** | | | | | | | | | | |
| Measure | D1 | D2 | D3 | D4 | D5 | D6 | D7 | D8 | D9 | D10 |
| Mean Theta | -1.833 | -1.036 | -0.607 | -0.337 | -0.083 | 0.184 | 0.459 | 0.759 | 1.11 | 1.754 |
| Mean test length | 8.37 | 7.56 | 7.79 | 7.83 | 7.93 | 7.95 | 8.01 | 8.27 | 8.56 | 13.02 |
| Mean standard error | 0.292 | 0.291 | 0.288 | 0.289 | 0.288 | 0.289 | 0.289 | 0.288 | 0.291 | 0.293 |
| Proportion stop rule satisfied | 100% | 100% | 100% | 100% | 100% | 100% | 100% | 100% | 100% | 96% |
| **Results at SEM 0.387** | | | | | | | | | | |
| Mean Theta | -1.833 | -1.036 | -0.607 | -0.337 | -0.083 | 0.184 | 0.459 | 0.759 | 1.11 | 1.754 |
| Mean test length | 4.74 | 4.85 | 4.85 | 4.89 | 4.94 | 5.01 | 5.03 | 5.26 | 5.46 | 6.21 |
| Mean standard error | 0.369 | 0.364 | 0.368 | 0.369 | 0.369 | 0.368 | 0.372 | 0.368 | 0.37 | 0.369 |
| Proportion stop rule satisfied | 100% | 100% | 100% | 100% | 100% | 100% | 100% | 100% | 100% | 100% |
| **Results at SEM 0.521** | | | | | | | | | | |
| Mean Theta | -1.833 | -1.036 | -0.607 | -0.337 | -0.083 | 0.184 | 0.459 | 0.759 | 1.11 | 1.754 |
| Mean test length | 2.7 | 2.84 | 2.94 | 2.94 | 3.1 | 3.04 | 3.2 | 3.31 | 3.44 | 3.89 |
| Mean standard error | 0.477 | 0.471 | 0.473 | 0.476 | 0.473 | 0.482 | 0.478 | 0.479 | 0.48 | 0.479 |
| Proportion stop rule satisfied | 100% | 100% | 100% | 100% | 100% | 100% | 100% | 100% | 100% | 100% |
