# Supplementary material for: Establishing language and ethnic equivalence for health-related quality of life item banks and testing their efficiency via computerised adaptive testing simulations
Source: PLoS One. 2024 Feb 23;19(2):e0298141. doi: 10.1371/journal.pone.0298141 (PMC10890744; doi:10.1371/journal.pone.0298141)
Supplement: S3 Table — (DOCX) [file pone.0298141.s003.docx]

| **S3 Table.** Simulation results for ***Social Relationship*** at three different precision stopping rule estimates across deciles of participant ability level | | | | | | | | | | |
| --- | --- | --- | --- | --- | --- | --- | --- | --- | --- | --- |
| **Results at SEM 0.3** | | | | | | | | | | |
| Measure | D1 | D2 | D3 | D4 | D5 | D6 | D7 | D8 | D9 | D10 |
| Mean Theta | -1.833 | -1.036 | -0.607 | -0.337 | -0.083 | 0.184 | 0.459 | 0.759 | 1.11 | 1.754 |
| Mean test length | 17.52 | 13.88 | 13.6 | 14.48 | 12.77 | 12.28 | 12.27 | 13.42 | 16.28 | 18.45 |
| Mean standard error | 0.295 | 0.294 | 0.293 | 0.293 | 0.292 | 0.291 | 0.292 | 0.293 | 0.293 | 0.295 |
| Proportion stop rule satisfied | 100% | 100% | 100% | 100% | 100% | 100% | 100% | 98% | 98% | 100% |
| **Results at SEM 0.387** | | | | | | | | | | |
| Mean Theta | -1.833 | -1.036 | -0.607 | -0.337 | -0.083 | 0.184 | 0.459 | 0.759 | 1.11 | 1.754 |
| Mean test length | 9.23 | 7.98 | 7.68 | 7.98 | 7.3 | 7.18 | 7.11 | 7.36 | 8.19 | 9.53 |
| Mean standard error | 0.377 | 0.374 | 0.374 | 0.374 | 0.374 | 0.373 | 0.376 | 0.376 | 0.376 | 0.38 |
| stop rule satisfied | 100% | 100% | 100% | 100% | 100% | 100% | 100% | 100% | 100% | 100% |
| **Results at SEM 0.521** | | | | | | | | | | |
| Mean Theta | -1.833 | -1.036 | -0.607 | -0.337 | -0.083 | 0.184 | 0.459 | 0.759 | 1.11 | 1.754 |
| Mean test length | 4.14 | 4.04 | 4.03 | 4.05 | 4 | 4 | 4 | 4.02 | 4.24 | 4.37 |
| Mean standard error | 0.503 | 0.487 | 0.483 | 0.486 | 0.481 | 0.48 | 0.483 | 0.486 | 0.487 | 0.499 |
| Proportion stop rule satisfied | 100% | 100% | 100% | 100% | 100% | 100% | 100% | 100% | 100% | 100% |
