# Supplementary material for: Establishing language and ethnic equivalence for health-related quality of life item banks and testing their efficiency via computerised adaptive testing simulations
Source: PLoS One. 2024 Feb 23;19(2):e0298141. doi: 10.1371/journal.pone.0298141 (PMC10890744; doi:10.1371/journal.pone.0298141)
Supplement: S4 Table — (DOCX) [file pone.0298141.s004.docx]

| **S4 Table.** Simulation results for ***Positive mindset*** at three different precision stopping rule estimates across deciles of participant ability level | | | | | | | | | | |
| --- | --- | --- | --- | --- | --- | --- | --- | --- | --- | --- |
| **Results at SEM 0.30** | | | | | | | | | | |
| Measure | D1 | D2 | D3 | D4 | D5 | D6 | D7 | D8 | D9 | D10 |
| Mean Theta | -1.833 | -1.036 | -0.607 | -0.337 | -0.083 | 0.184 | 0.459 | 0.759 | 1.11 | 1.754 |
| Mean test length | 32.1 | 30.75 | 26.68 | 20.39 | 17.03 | 17.13 | 17.83 | 18.84 | 17.34 | 17.91 |
| Mean standard error | 0.307 | 0.302 | 0.298 | 0.297 | 0.296 | 0.295 | 0.294 | 0.295 | 0.296 | 0.295 |
| Proportion stop rule satisfied | 35% | 63% | 98% | 99% | 99% | 100% | 100% | 100% | 98% | 98% |
| **Results at SEM 0.387** | | | | | | | | | | |
| Mean Theta | -1.833 | -1.036 | -0.607 | -0.337 | -0.083 | 0.184 | 0.459 | 0.759 | 1.11 | 1.754 |
| Mean test length | 18.4 | 15.89 | 11.76 | 9.72 | 9.99 | 10.63 | 9.92 | 10.25 | 9.73 | 9.95 |
| Mean standard error | 0.382 | 0.383 | 0.382 | 0.376 | 0.378 | 0.376 | 0.374 | 0.375 | 0.377 | 0.376 |
| stop rule satisfied | 100% | 100% | 100% | 100% | 100% | 100% | 100% | 100% | 100% | 100% |
| **Results at SEM 0.521** | | | | | | | | | | |
| Mean Theta | -1.833 | -1.036 | -0.607 | -0.337 | -0.083 | 0.184 | 0.459 | 0.759 | 1.11 | 1.754 |
| Mean test length | 7.59 | 6 | 4.69 | 5.22 | 5.12 | 4.83 | 4.77 | 4.97 | 4.95 | 5.02 |
| Mean standard error | 0.511 | 0.511 | 0.507 | 0.501 | 0.502 | 0.5 | 0.497 | 0.498 | 0.493 | 0.5 |
| Proportion stop rule satisfied | 100% | 100% | 100% | 100% | 100% | 100% | 100% | 100% | 100% | 100% |
